# The association between drug pricing and drug shortage in Saudi Arabia: a retrospective database analysis

**DOI:** 10.1186/s40545-023-00591-8

**Published:** 2023-07-18

**Authors:** Mohammad A. Alowairdhi, Fatimah H. Alhussain, Maha I. Alomran, Ohoud A. Almadani, Norah A. Alkhammash, Shayma F. Alyabes, Sultan A. Alharbi, Esraa A. Almudaiheem, Reem A. Alhaider, Sara A. Alhassan, Zainab A. Almuallem, Nuha Algain, Abdulaziz O. Alshehri, Thamir M. Alshammari, Turki A. Althunian

**Affiliations:** 1 Department of Pricing and Pharmacoeconomics, Saudi Food and Drug Authority, Riyadh, Saudi Arabia; 2 Research Informatics Department, Saudi Food and Drug Authority, Riyadh, Saudi Arabia; 3 Drug Availability and Tracking Center, Saudi Food and Drug Authority, Riyadh, Saudi Arabia; 4 College of Pharmacy, Riyadh Elm University, Riyadh, Saudi Arabia; 5 College of Medicine, Alfaisal University, Riyadh, Saudi Arabia

**Keywords:** Drug shortage, Pricing, Regulatory science

## Abstract

**Background:**

Previous studies have suggested that drug pricing could contribute to drug shortages; however, there is limited quantitative assessment of this potential causal association. This retrospective database analysis aimed to investigate the association between drug prices and drug shortage incidents in Saudi Arabia.

**Methods:**

This was a retrospective database analysis study. Drugs with shortage notifications sent to the Saudi Food and Drug Authority (SFDA) between January 2017 and December 2020 were included. Each drug's foreign-to-Saudi price ratio (FTSPR) was calculated by dividing the mean international price by the Saudi price. Drugs were categorized into three groups based on their FTSPR: Group 1 (FTSPR > 1), Group 2 (FTSPR = 1), and Group 3 (FTSPR < 1). The primary outcome was the ratio of mean counts (mCR) between the three groups, with Group 3 serving as the control group. The analysis was adjusted for the measured confounders using a negative binomial regression model.

**Results:**

A total of 900 drugs were included in the study, with 348 in Group 1, 345 in Group 2, and 209 in Group 3. The mean count in Group 1 was higher compared to Group 3 (mCR: 1.88; 95% confidence interval [CI] 1.24 to 2.83), while the mean counts between Group 2 and Group 3 were comparable (mCR: 1.39; 95% CI 0.92 to 2.09).

**Conclusions:**

Our findings indicate an association between drug shortage incidents and higher prices of drugs outside Saudi Arabia. Further studies are needed to explore this causal relationship in different contexts.

## Background

Drug shortage was defined by the United States [US] Federal Food, Drug, and Cosmetic Act as periods when the demand or projected demand for a drug exceeds the supply [1]. It has emerged as a significant global challenge due to its multifaceted nature and the adverse clinical, social, and economic consequences it entails [1–21]. A recent study found that a large percentage of drug shortage incidents in the Netherlands were anticipated to have moderate to high negative clinical, humanistic or economic patient impacts [22]. Various studies have identified clinical, economic, regulatory, and policy-related factors as contributors to drug shortages [1–21]. Among these factors, drug pricing has been identified as a potential risk; however, only a few studies have explored this causal association, and none was conducted in Saudi Arabia [1–21].

An analysis of a US database of commercial outpatient pharmacy claims, period 2008 to 2014, revealed that the duration of drug shortages was influenced by the gradual percentage increase in drug prices (i.e. higher percentage increases were associated with longer durations of drug shortages) [11]. However, the study solely focused on generic drugs [11]. A Canadian study examining a new generic pricing strategy implemented in 2014, known as the Tiered-Pricing Framework, did not find an association with the risk of disrupting the entry of generic drugs into Canada (although this study did not analyze actual shortage incidents) [20]. Other studies indirectly addressed this causal association or considered it as a secondary outcome (e.g. a Canadian study suggested that markets with a single generic manufacturer and lower profit margins were more susceptible to shortages) [21].

Limited research exists in Saudi Arabia regarding drug shortages in general and on the identification of risk factors related to such shortages in particular [3, 14–16]. Two studies have touched upon drug pricing as a potential risk factor for drug shortages, albeit without conducting quantitative causal analyses [15, 16]. The pricing of both prescription and non-prescription drugs in Saudi Arabia is determined and regulated by the Saudi Food and Drug Authority (SFDA) in accordance with the SFDA pharmaceutical pricing guideline [23]. Upon receiving initial marketing authorization approval from the SFDA, brand-name small molecules, biologics, and generic drugs intended for the public and private sectors in Saudi Arabia are subject to the SFDA pricing system [23]. The initially assigned price undergoes a price-revaluation process throughout its regulatory life cycle, allowing for a maximum reduction of 30% under specific circumstances such as revising the prices of the entire therapeutic class, lowering the price in the manufacturer's country, renewing the marketing authorization, or modifying the status of the marketing authorization (i.e., variation) [23]. This study aimed to evaluate the association between drug prices and the likelihood of drug shortage incidents using a Saudi drug shortage population.

## Methods

### Study design and data source

To identify drugs reported as experiencing shortages between January 2017 and December 2020, data were extracted from the database maintained by the Saudi Food and Drug Authority’s (SFDA) Drug Availability and Tracking Center. Various stakeholders, including marketing authorization holders, public and private health sectors, and patients, utilize different reporting systems such as the Track and Trace System to report incidents of drug shortages to the SFDA, usually referencing the SFDA’s drug registration number [23]. However, in order to validate the occurrence of an actual drug shortage incident, confirmation from the SFDA (i.e., validation process) is required. In the study period, confirmation of the reported shortage incident relied on the marketing authorization holder. Presently, the confirmation of the reported shortage incident is accomplished by cross-referencing the SFDA Track and Trace System and/or verifying the existence of drug shortages with the marketing authorization holder.

All identified shortage incidents were unique for each SFDA drug registration number, meaning that no duplicate shortage incidents were found. The inclusion criteria did not consider the severity or anticipated negative clinical impact of the shortage. Once drugs with shortage incidents were identified, their local and international prices at the time of the shortage report were obtained from the SFDA database and the S&P Global Database [24]. The latter is an information service provider that extracts the updated international prices from the authenticated and relevant sources within each country [24]. Relevant data regarding potential confounding factors were extracted from both the internal and publicly available domains of the SFDA.

### Study exposures, outcomes and confounders

For each drug, whether it was a brand-name small molecule, biologic or generic drug, the study calculated the foreign-to-Saudi price ratio (FTSPR) by dividing the mean price of the drug in the international market (numerator) by its price in Saudi Arabia (denominator) during the year of the shortage incident. Based on the FTSPR ratio, the drugs were classified into three groups: Group 1 included drugs with higher international prices compared to local prices (FTSPR ratio > 1), Group 2 included drugs with similar international and local prices (FTSPR ratio = 1), and Group 3 included drugs with higher local prices compared to international prices (FTSPR ratio < 1). Group 3 was chosen as the control arm for between-group comparisons, assuming that the likelihood of shortage due to pricing might be lower in this group given their high local prices.

The analysis of the study outcome was adjusted for several confounding variables, including the type of drug registration (brand-name small molecules, biologics, local, or international generics), route of administration (oral, injections, or other preparations), country of the manufacturer (local, regional, or international companies), cause of the shortage (as outlined in Table 1), and whether the shortage incident was reported before or after the confirmation of human-to-human transmission of coronavirus disease 2019 (COVID-19) (pre-2020 vs. 2020). The selection of these confounding variables was based on direct acyclic graphs (DAGs) and previous literature indicating their potential as risk factors for drug shortages [1–21].

**Table 1:** Description of causes of drug shortage

Cause of shortage	Definition
Marketing challenges	Low demands, contract terminations, etc.
Voluntary discontinuation	The marketing authorization holder submitted a request for drug marketing discontinuation
Manufacturing difficulties	Obstacles to manufacturing processes (e.g. machinery malfunction, shortage in the supply of active pharmaceutical ingredients, concerns related to the quality of the manufacturing, etc.)
Regulatory actions	Incompliance with the SFDA regulations

### Statistical analysis

Descriptive summaries were provided for the study groups, and Chi-square tests were used to explore differences in the distribution of the confounding variables among the groups. The main hypothesis of the study was that the number of shortage incidents in drugs that are more expensive in Saudi Arabia (Group 3) would be lower compared to the other groups. To test this hypothesis, mean count ratios (mCRs) were estimated between Group 1 vs. Group 3 and Group 2 vs. Group 3. A negative binomial regression model was employed to estimate the mCRs and their corresponding 95% confidence intervals while adjusting for the measured confounding variables. The use of a Poisson regression model was not feasible due to the presence of overdispersion in the data. All analyses were performed using RStudio Version 1.2.5033.

## Results

During the study period, a total of 1082 drugs were reported to the SFDA as experiencing shortages, resulting in an average rate of 271 shortage incidents per year. The highest number of shortages occurred in 2019 (n = 544) and 2020 (n = 300). Pricing details necessary for calculating the FTSPR were available for 900 out of the 1082 drugs, representing 427 unique active pharmaceutical ingredients or fixed combinations. The median duration of shortage was 8.2 months, excluding drugs that were discontinued from the market and those reported in 2017 (the year during which the shortage notification system was established).

**Fig. 1:**
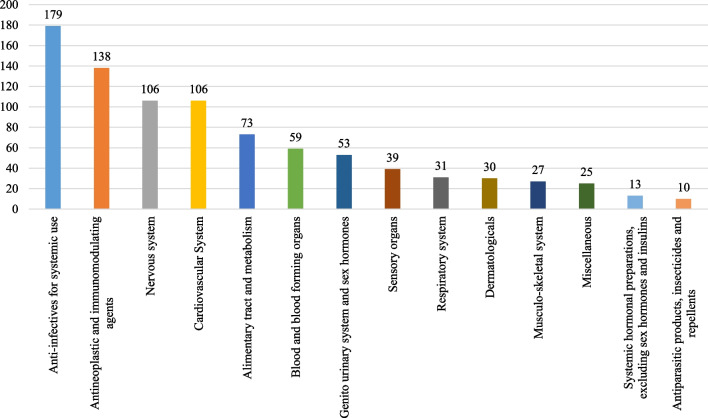
The most commonly reported therapeutic classes with shortage

Among the 900 drugs with available pricing details, the majority were brand-name small molecules (47%) and international generics (38%) (Table 2). In terms of preparations, 47% were oral and 40% were injectable. The most common reasons for shortage were voluntary discontinuation of registrations by the marketing authorization holders (47.5%) and marketing challenges (23.2%). Additionally, the majority of shortages occurred prior to the confirmation date of human-to-human transmission of COVID-19 (Table 2). The shortages encompassed 13 main therapeutic classes based on the Anatomical-Therapeutic Chemical (ATC) classification system maintained by the World Health Organization, as depicted in Fig. 1. The most frequently reported drugs in shortage were anti-infectives for systemic use (19.9% of the 900 drugs) and antineoplastic and immunomodulating agents (15.3% of the 900 drugs). Notable examples of commonly reported drugs in shortage included temozolomide (n = 15), glucose/dextrose (n = 13), cephalexin (n = 13), methotrexate (n = 12), and warfarin (n = 11).

**Table 2:** Characteristics of the study groups

Study confounders	Study groups			P-values
	Group 1 (FTSPR ratio > 1)	Group 2 (FTSPR ratio = 1)	Group 3 (FTSPR ratio < 1)	
	N = 348 (%)	N = 343 (%)	N = 209 (%)	
Type of drug registration				
Brand-name small molecules	181 (52)	149 (43)	91 (44)	< 0.01
Biologics	19 (5.5)	5 (1)	4 (2)	
International generics	122 (35)	136 (40)	86 (41)	
Local generics	26 (7.5)	53 (16)	28 (13)	
Route of administration				
Oral preparations	145 (41.5)	160 (47)	114 (55)	< 0.01
Injections	141 (40.5)	149 (43)	69 (33)	
Other preparations (eye drops, creams, lotions, suppositories, etc.)	62 (18)	34 (10)	26 (12)	
Country of the manufacturer				
International	278 (80)	251 (73)	151 (72.3)	< 0.01
Local	31 (9)	67 (20)	32 (15.3)	
Regional	39 (11)	25 (7)	26 (12.4)	
Cause of shortage				
Marketing challenges	98 (28)	60 (17)	51 (24.4)	
Voluntary discontinuation	120 (34)	212 (62)	94 (45)	< 0.01
Manufacturing difficulties	100 (29)	52 (15)	51 (24.4)	
Regulatory actions	30 (9)	19 (6)	13 (6.2)	
Reporting relevant to COVID19				
Before COVID19	212 (61)	252 (73)	136 (65)	< 0.01
After COVID19	136 (39)	91 (27)	73 (35)

Most of the reported drugs with shortage during the study period belong to Group 1 (FTSPR > 1, n = 348), followed by drugs in Group 2 (FTSPR = 1, n = 343), and Group 3 (FTSPR < 1, n = 209). According to the negative binomial regression model, drug shortages were higher in Group 1 compared with Group 3 (mCR: 1.88; 95% confidence interval [CI] 1.24 to 2.83). However, the mean counts of between Group 2 and Group 3 were comparable, with an mCR of 1.39 (95% CI 0.92 to 2.09).

## Discussion

Our study results demonstrated that drug shortages were mostly reported for drugs with higher prices in markets outside of Saudi Arabia. This group of drugs was also more likely to be associated with shortage incidents compared to drugs with higher prices in Saudi Arabia. The likelihood of shortage was found to be similar between drugs with comparable prices both inside and outside of Saudi Arabia, as well as drugs with higher prices in Saudi Arabia.

Our study revealed a potential association between drug pricing and the risk of shortage, which aligns with findings from a previous analysis of a US database of commercial outpatient pharmacy claims spanning from 2008 to 2014 [11]. To the best of our knowledge, these two studies are the only available literature that quantitatively examine the relationship between drug pricing and the risk of shortage. It is important to note that the majority of shortage incidents included in our study occurred prior to the implementation of the updated pricing guideline in late 2020 (the guideline was officially published in early 2021) [23]. One of the main objectives of updating the guideline was to mitigate the risk of drug shortage during pricing processes, such as by reviewing the availability of alternative treatment options and adjusting price-related requests in a manner that does not disrupt product supply [23]. A future study should be conducted to evaluate the impact of this guideline update on the likelihood of drug shortages. Such studies may assist policy makers in incorporating drug availability considerations into the pricing mechanisms of pharmaceutical products.

In contrast to the United States and Europe, our study found that the most commonly reported therapeutic classes experiencing shortages in Saudi Arabia were anti-infectives for systemic use and antineoplastic and immunomodulating agents. In the US and Europe, the most commonly reported therapeutic classes with shortages were drugs for the central nervous system, followed by fluids/electrolytes in the US and drugs for the cardiovascular system in Europe [11, 17, 19, 25]. The number of shortage incidents in 2020 in Saudi Arabia (n = 300) was lower compared to the US (n = 1106), Spain (n = 814), Norway (n = 800), Sweden (n = 890), and Finland (n = 1522) [19]. The duration of shortages observed in our study was comparable to the reported shortage duration in the US (8.2 months vs. 8.4 months, respectively) [11]. In our study, less than half of the drugs experiencing shortages were discontinued voluntarily by the marketing authorization holders, with reasons for discontinuation not provided in the system. The second most commonly reported reason for shortage was marketing challenges. On the other hand, supply/demand issues (where manufacturers are unwilling or unable to meet drug demand) and manufacturing issues (such as quality problems) were the most commonly reported reasons for shortage in the US [17, 26]. A survey conducted by the European Association of Hospital Pharmacists in 2019 indicated that global shortages of active pharmaceutical ingredients and manufacturing problems were the most frequently cited possible reasons for drug shortages [18]. Furthermore, similar to Europe, our study found that oral preparations were more likely to experience shortages, while in the US, injectable preparations were more prone to shortages [11, 17, 19, 26].

Our study represents the first analysis to investigate the causal association between drug prices, or any other risk factor for drug shortage, and the likelihood of drug shortage in Saudi Arabia. It is also one of the few studies in the literature that quantitatively assessed this causal relationship. All shortage notification data in our study underwent validation by the SFDA upon receiving a notification; thereby enhancing the validity of the study data. However, our study has two limitations. Firstly, the reasons behind the voluntary discontinuation of marketing authorization for less than half of the studied drugs remain unknown. Secondly, due to the recent establishment of the Drug Availability and Tracking Center at that time, complete availability of the 2017 and 2018 drug shortage notifications was not achieved.

## Conclusions

Our study found that drug pricing may be associated with the risk of shortage, with drugs that have higher prices outside Saudi Arabia being more likely to be in shortage. The study also highlights the need for further research on the impact of drug pricing on the risk of drug shortages and the need for measures to address the various reasons for drug shortages.

## Data Availability

The datasets generated and/or analysed during the current study are not publicly available due confidentiality but are available from the corresponding author on reasonable request (some data are available from S&P Global Database but restrictions apply to the availability of these data, which were used under license for the current study, and so are not publicly available).

## Abbreviations

US: United States
SFDA: Saudi Food and Drug Authority
FTSPR: Foreign-to-Saudi price ratio
COVID-19: Coronavirus disease 2019
DAGs: Direct acyclic graphs
mCR(s): Mean count ratio(s)
ATC: Anatomical-Therapeutic Chemical
CI: Confidence interval
